# Medical Institute of Louisville

**Published:** 1841-11

**Authors:** 


### MEDICAL INSTITUTE OF LOUISVILLE.

The *fifth* class is now assembling at this institution, and promises to be larger than any preceding one. Already about 200 students have reached the city, and if the proportion behind is as large as usual, the class will no doubt reach 250, by the first of December. This is most encouraging success, and must be exceedingly gratifying to the friends of the institution. No Medical School in the country has grown with such rapidity, and with the excellent facilities it possesses for imparting a thorough practical knowledge of medicine, it can hardly fail to maintain a high rank among the American Schools. Valuable additions were made to the library and apparatus, by Professor Caldwell, during his recent visit to Europe, all of which were received before the opening of the present course.

